# Convincing 10-Year Follow-up Results of the Banded Roux-en-Y Gastric Bypass

**DOI:** 10.1007/s11695-024-07113-8

**Published:** 2024-02-23

**Authors:** Marijn T. F. Jense, Nina Meuwissen, Abdelrahman M. Galal, Evelien De Witte, Sofie Fransen, Pieter P. H. L. Broos, Jan Willem M. Greve, Evert-Jan G. Boerma

**Affiliations:** 1 Bariatric Surgery at Zuyderland Medical Center, Henri Dunantstraat 5, 6419 PC Heerlen, The Netherlands; 2 General Surgery Department, Sohag Faculty of Medicine, Sohag University Hospitals, Sohag, Egypt; 3 Dutch Obesity Clinic South, John F. Kennedylaan 301, 6419 XZ Heerlen, The Netherlands; 4 Research Institute NUTRIM, Faculty of Health, Medicine and Life Sciences at Maastricht University, Maastricht, The Netherlands; 5 Faculty of Health Medicine and Life Sciences, Maastricht University, Maastricht, The Netherlands

**Keywords:** Bariatric surgery, Roux-en-Y gastric bypass, Silicone banding, Banded gastric bypass, Obesity, Weight loss, Long-term results

## Abstract

**Introduction:**

Several studies have shown the positive effect on weight loss of the banded Roux-en-Y gastric bypass (BRYGB). Thus far, studies describing the 10-year post-operative results are scarce. Therefore, the aim of this study was to describe the weight loss results, effect on associated medical problems, and complication rates during 10 years of follow-up after BRYGB.

**Method:**

Data were collected from patients who underwent laparoscopic BRYGB with a non-adjustable silicone gastric ring between January 2011 and March 2013. All patients were included when found to be eligible according to the IFSO criteria.

**Results:**

One hundred forty-nine patients were included, 110 received a primary BRYGB and 39 received a conversional BRYGB. The primary BRYGB group consisted of 68% female patients with a mean BMI of 44.5 kg/m^2^ and a mean age of 46 years old. The conversional group consisted of 77% females and had a mean BMI of 34.8 kg/m^2^ and a mean age of 48 years. At 10-year follow-up, 67.1% of the data was available. Ten-year post-operative 30% total weight loss was seen in the primary group, and 7% in the conversional group. In 10 years, 23% of the patients had complications of which half were ring-related.

**Conclusion:**

The addition of a silicon ring to the Roux-en-Y gastric bypass may result in substantial and stable weight loss maintenance 10 years post-operative. Furthermore, the number of patients with long-term complications was low and the number of associated medical problems was significantly reduced.

**Graphical Abstract:**

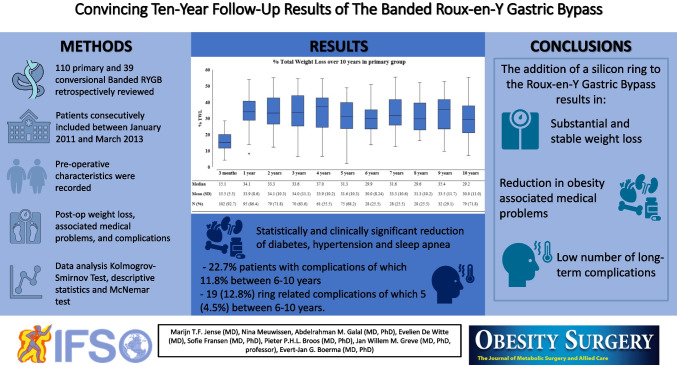

## Introduction

The most effective long-term treatment for people with obesity is bariatric surgery. The Roux-en-Y gastric bypass (RYGB) is considered the most effective treatment by many [1–3]. However, even with the RYGB, weight recurrence or insufficient weight loss can occur [4]. Optimization of any bariatric treatment should therefore be thoroughly investigated. One possible method of optimizing the RYGB is by adding a non-adjustable ring on the pouch, creating a banded Roux-en-Y gastric bypass (BRYGB).

However, the BRYGB remains to be a controversial procedure for many. Even though multiple studies have shown better weight loss results than the regular RYGB [5, 6]. Some surgeons remain reluctant to use a silicone ring because of the possible risk of ring-related complications, even though a number of studies have shown a low risk of 0–4% of ring-related complications [5–8]. The exact effect of the ring is yet unknown; however, the hypothesis is that with the ring earlier satiety is obtained. When the pouch is filled, the ring gives pressure on the gastric wall creating the feeling of satiety. When the pouch is not filled, the ring does not touch the pouch and does therefore not cause restriction.

In several publications, 5-year follow-ups of BRYGB and non-BRYGB patients have been reported [5, 7]. These papers demonstrated a higher weight loss result in the BRYGB group while showing similar complication rates among both groups. Although these results are promising, few studies have described the results of the BRYGB with a follow-up of more than 5 years. Studies that do describe data exceeding 5 years usually have low follow-up rates and, therefore, reflect a less representable image of the treated population [9]. To obtain more information about the long-term follow-up results of the BRYGB, patients with a follow-up period of 10 years were studied.

The primary aim of this study was to describe the weight loss results 10 years after a BRYGB. As secondary objectives, this study aimed to describe the long-term effect of a BRYGB on the following associated medical problems: type 2 diabetes, hypertension, obstructive sleep apnea syndrome (OSAS), and dyslipidemia; and to describe (ring-related) complication rates. This study is a follow-up study of a previously published study with the 5-year results of the same group of patients [7].

## Methods

Data were collected from consecutive patients who underwent a laparoscopic BRYGB with a non-adjustable silicone gastric ring in the Zuyderland Medical Center (ZMC) either as a primary or conversional operation between January 2011 and March 2013. Local approval for data collection was obtained from the ethics committee in accordance with the ethical standards as laid down in the 2013 Declaration of Helsinki. The 5-year post-operative results have already been described in an article by Galal et al. [7]. In this study, the focus will be on the 6–10-year post-operative results of the same patient population.

### Eligibility

All included patients were intensively screened for eligibility for surgery. Eligible patients for a primary procedure were between 18 and 65 years old and had a BMI > 40 kg/m^2^ or > 35 kg/m^2^ with medical problems associated with obesity. For patients who received the BRYGB surgery as revisional surgery, there were no requirements considering BMI or age. Patients who received RYGB surgery with an adjustable band or an open BRYGB surgery were excluded from this study.

### Surgical Procedure

All participants underwent a laparoscopic Roux-en-Y gastric bypass (RYGB). The pouch with a length of 6–8 cm is calibrated on a 40 French orogastric tube. After the creation of the pouch, two limbs were formed: a biliopancreatic limb with a length of 60 cm and an alimentary limb with a length of 120 cm. The gastrojejunostomy and jejunojejunostomy were created using a linear stapler for the anastomosis and suture closure of the remaining defect. Finally, 1–2 cm above the gastrojejunostomy, a silicone ring was placed. Patients received a non-adjustable silicone ring with a size between 6 and 8 cm. Initially, the standard ring size was 6 cm for females and 6.5 cm for males and conversional surgery. This was changed to the standard ring size of 6.5 cm for females and 7.0 cm for males and conversional surgery because of early dysphagia. A medical-grade ventriculoperitoneal drain was used to create the ring.

### Treatment

All patients followed the standard 5-year follow-up treatment by a multidisciplinary team, consisting of a dietician, psychologist, exercise therapist, medical doctor, and surgeon at the Dutch Obesity Clinic (DOC, Nederlandse Obesitas Kliniek). The follow-up treatment consisted of a consultation by phone 1 week and 6 weeks post-operative and seven visits at the DOC at 3 months, 6 months, 12 months, 24 months, 36 months, 48 months, and 60 months. During these appointments, weight, BMI, medication use, obesity-related co-morbidities, and procedure-related complications and surgical interventions were recorded.

### Loss to Follow-up

Data of almost all patients was available until the 5-year follow-up appointment. Data after this appointment was either extracted from the patient files from the DOC or ZMC or the patient was contacted via telephone to obtain the missing data directly from the patient. If not available from either the patient file or through the telephone call, the data was considered missing and recorded as such.

### Weight

At every follow-up consultation at the DOC, weight was measured. After 5 years, the regular follow-up visits were transferred to the general practitioner of the patient. Weight after this date was recorded using the electronic patient file of the hospital. After 10 years, all patients were contacted by telephone to obtain their weight, if weight was not available in the electronic patient file of either the ZMC or the DOC. Weight loss was expressed using percentage total weight loss (%TWL). Weight recurrence was measured using the lowest weight during follow-up and the weight at either 5- or 10-year follow-up.

### Associated Medical Problems

The following obesity-associated medical problems were studied*:* type 2 diabetes, obstructive sleep apnea syndrome (OSAS), hypercholesterolemia, and hypertension. For each associated medical problem, the status 10 years after surgery was compared to the status at the pre-surgical baseline measurement. The definition of associated medical problems of the ASMBS was used as a guideline [10].

### Complications

All complications related to the surgery were recorded in the patient file in the hospital. For analysis of these complications after 10 years, this data was collected and added to the existing 5-year follow-up dataset. All complications were scored using the Clavien-Dindo classification system and were separated into early (i.e., < 30 days post-operative) or late (i.e., > 30 days post-operative) complications and into regular or ring-related complications.

### Statistical Analysis

All statistical tests were performed using IBM SPSS statistics for Windows, version 26, Armonk, NY. Using the Kolmogorov–Smirnov test, all results were tested for normality. All continuous data are expressed as means and standard variations. The categorical variables are expressed as percentages for the baseline measurement and 5 years and 10 years after surgery.

Differences in the prevalence of associated medical problems at different time points compared to baseline were assessed using the McNemar test.

The participants were divided into two separate groups. Conversional surgery patients were analyzed separately from the primary patients. A *p*-value of 0.05 was considered statistically significant.

## Results

### Baseline Characteristics

From our total study population of 149 patients, 110 had a primary BRYGB and 39 a conversional operation. The primary BRYGB group consisted of 68% female patients with a mean BMI of 44.5 kg/m^2^ and a mean age of 46 years (Table 1). The conversional group consisted of 77% females and had a mean BMI of 34.8 kg/m^2^ and a mean age of 48 years. Reasons for conversional surgery were dysphagia (61.5%), weight recurrence (25.6%), insufficient weight loss (7.7%), pouch dilatation (2.6%), and staple line dehiscence (2.6%). The time between the primary and conversional surgery was 129.7 (± 76.7) months on average with a minimum of 14 months and a maximum of 288 months. From the 39 conversional surgeries performed, 28 (71.8%) were vertical banded gastroplasty, 7 (17.9%) were sleeve gastrectomy, 3 (7.7%) were adjustable gastric band, and 1 (2.6%) was a RYGB.

**Table 1:** Baseline characteristics

*Baseline characteristics*		*Total (N = 149)*	*Primary (N = 110)*	*Conversional (N = 39)*
*Sex*	Female (%)	105 (70.5)	75 (68.2)	30 (76.9)
*Mean age on day of operation in years (SD)*		47 (11.4)	46 (12)	48 (10)
*Mean weight screening in kg (SD)*		120.3 (26.8)	127.5 (23.0)	100.1 (26.8)
*Mean BMI screening in kg/m*^*2*^* (SD)*		42.0 (8.4)	44.5 (6.9)	34.8 (8.0)
*Diabetes (%)*		77 (51.7)	68 (61.8)	9 (23.1)
*Hypertension (%)*		66 (44.3)	55 (50)	11 (28.2)
*OSAS (%)*		35 (23.5)	32 (29.1)	3 (7.7)
*Dyslipidemia (%)*		38 (25.5)	37 (33.6)	1 (2.6)

*SD* standard deviation, *OSAS* obstructive sleep apnea syndrome

### Loss to Follow-up

The percentage lost to follow-up differed per time point, ranging from 25.5 to 92.7% as presented below in all figures and tables. After the 10-year follow-up, data was available for 100/149 patients (67.1%). Forty-nine patients (32.9%) could not be reached. Of these 49 patients, 8 patients had died during the follow-up period. Their deaths were not related to the bariatric procedure. When dividing into primary and conversional groups, 31/110 patients (28.2%) from the primary group and 18/39 patients (46.2%) from the conversional group were lost to follow-up. At 10 years, 60% of the weight loss data was self-reported data, obtained via a telephone call.

### Ring Size

In the primary group, most patients had a ring size of 6.5 (48.2%), followed by 7.0 (21.8%) and 7.5 (20.9%). Only 9 patients (8.2%) had a ring size of 6.0 and 1 (0.9%) of 8.0. In the conversion group, 30 patients (76.9%) had a ring size of 7.0, 5 (12.8%) of 6.5, and 2 patients (5.1%) had 7.5, and 2 patients (5.1%) had a 8.0-cm ring.

### Weight

**Fig. 1:**
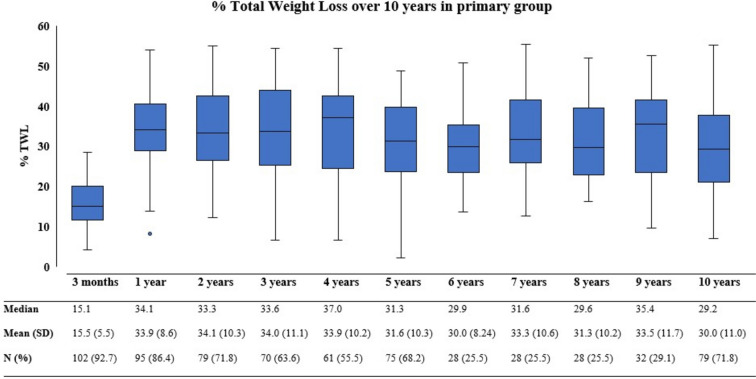
Boxplot graph presenting % total weight loss over 10 years in the primary group

**Fig. 2:**
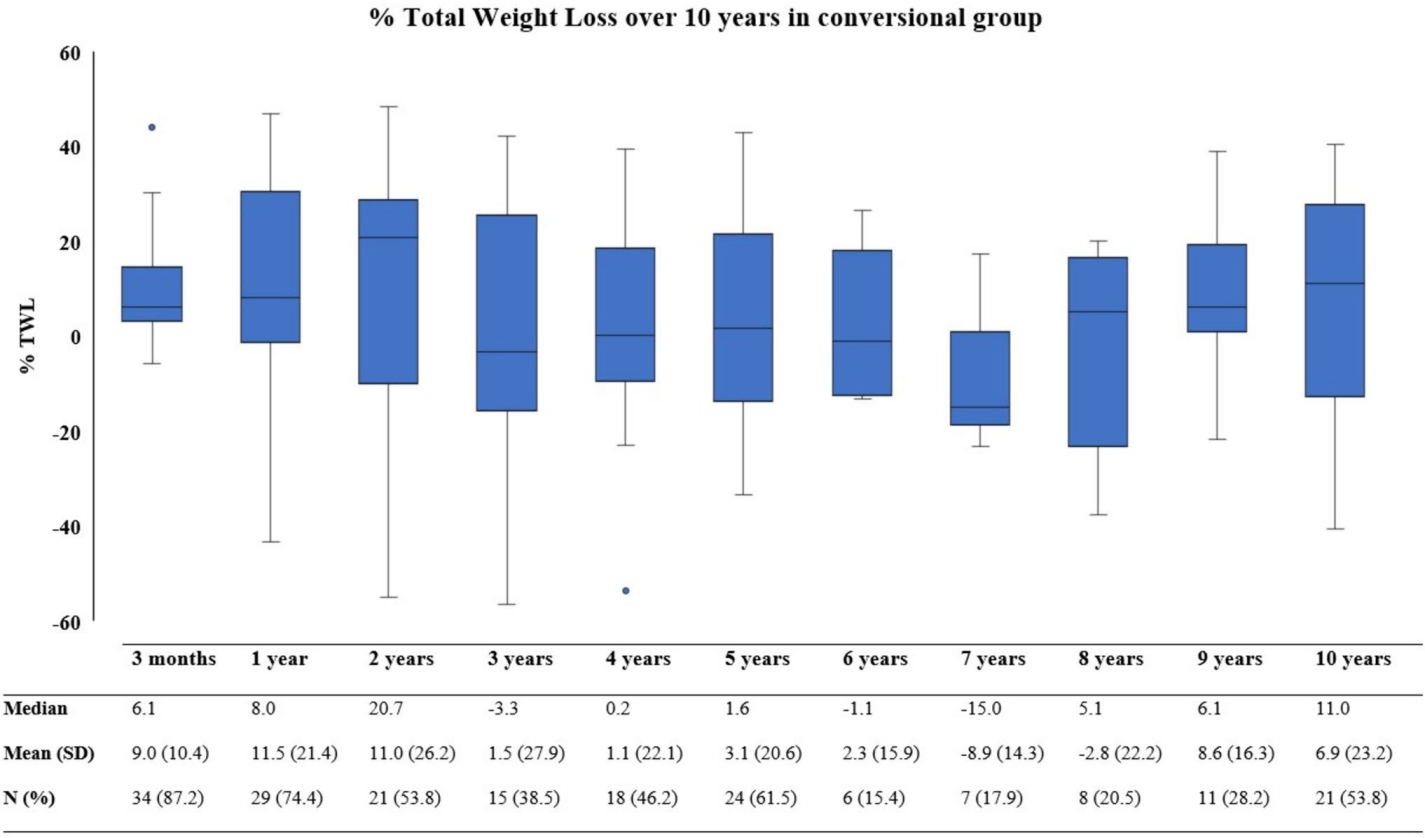
Boxplot graph presenting % total weight loss over 10 years in the conversional group

As presented in Fig. 1, the %TWL over time first increases up to a mean of 34.1% at 2 years which slightly decreases to 30.0% at 10 years post-operative in the primary group. Figure 2 displays the %TWL over time for the conversional group, with a mean %TWL of 6.9 at 10 years after revision. Table 2 displays the percentages of weight recurrence after 5 and 10 years post-operative. As one can see in these tables, weight recurrence increases slightly over time.

**Table 2:** Weight recurrence after 5 and 10 years in percentage of the lowest weight

**After 5 years**	**Primary** ***N* = 75**	**Conversional** ***N* = 24**
> 5% weight recurrence*	45.9%	83.3%
> 10% weight recurrence*	21.6%	66.7%
> 20% weight recurrence*	1.4%	20.8%
**After 10 years**	**Primary** ***N* = 79**	**Conversional** ***N* = 21**
> 5% weight recurrence*	65.8%	52.4%
> 10% weight recurrence*	29.1%	47.6%
> 20% weight recurrence*	5.1%	19.0%

^*^Based on the lowest weight between surgery and 5 and 10 years post-operative

### Associated Medical Problems

Table 3 presents the data on associated medical problems over the follow-up period. The number of patients suffering from hypertension and OSAS decreased and stayed low until at least 10 years after the operation. For DM and dyslipidemia, a decrease to 14.4% and 12.6% at 5 years compared to baseline incidence is visible with a slight increase compared to the 5-year results, but still a decrease compared to baseline at 10 years to 29.6% and 20% respectively. All values were statistically different compared with baseline values, except for dyslipidemia at 10 years post-operative.

**Table 3:** Presence of associated medical problems at 5 and 10 years post-operative

	**Primary RYGB**				
	**BMI (SD)**	**Diabetes mellitus**	**Hypertension**	**OSAS**	**Dyslipidemia**
Baseline	45 (7)	61.2% (*n* = 110)	50% (*n* = 110)	29% (*n* = 110)	34% (*n* = 108)
5 years	30.2 (5.2)	14.4%* (*n* = 97)	28.4%* (*n* = 102)	12%* (*n* = 100)	12.6%* (*n* = 103)
10 years	31.4 (6.7)	29.6%* (*n* = 71)	25%* (*n* = 68)	12%* (*n* = 66)	20.0% (*n* = 70)
	**Conversional RYGB**				
	**BMI (SD)**	**Diabetes mellitus**	**Hypertension**	**OSAS**	**Dyslipidemia**
Baseline	35 (8)	23.1% (*n* = 39)	28.2% (*n* = 39)	7.9% (*n* = 38)	2.7% (*n* = 37)
5 years	31.7 (6.5)	9.4% (*n* = 32)	18.8% (*n* = 32)	5.6% (*n* = 36)	2.8% (*n* = 36)
10 years	31.4 (8.8)	6.7% (*n* = 15)	14.3% (*n* = 14)	7.1% (*n* = 14)	14.3% (*n* = 14)

*RYGB* Roux-en-Y gastric bypass, *BMI* body mass index, *OSAS* obstructive sleep apnea syndrome

^*^Statistical significant difference compared with baseline (tested with McNemar’s test, with *a* = 0.05)

The remission rate in the primary group of DM was 75.9%, hypertension 42.9%, OSAS 52.0%, and dyslipidemia 60.6% at 5 years and 53.3%, 51.4%, 61.9%, and 46.2% at 10 years respectively. The remission rate in the conversional group of DM was 40%, hypertension 33.3%, OSAS 50%, and dyslipidemia 0% at 5 years and 0%, 60%, 75%, and 0% at 10 years respectively.

### Complications

All complications with a Clavien-Dindo score of 3 and higher are presented in Table 4 and 5. Table 5 represents the new data from 6 to 10 years post-operatively; Table 4 represents the total data. The complication rates of < 30 days post-operative are described in the paper by Galal et al. [7].

**Table 4:** Complications between more than 30 days and 10 years post-operative

Complications > 30 days–10 years		Primary (%)	Conversional (%)	Total (%)
Patients with complications		25 (22.7)	7 (17.9)	32 (21.5)
Total complications		46	11	57
Ring-related*		14 (12.7)	3 (7.6)	17 (11.4)
	*Erosion*	3 (2.7)	2 (5.1)	5 (3.4)
	*Dysphagia*	5 (4.5)	1 (2.6)	6 (4.0)
	*Slippage*	4 (3.6)	0	4 (2.7)
	*Stenosis*	1 (0.9)	0	1 (0.7)
	*Broken ring*	1 (0.9)	0	1 (0.7)
Ring-unrelated		32 (29.1)	8 (20.5)	40 (26.8)
	*Pouch revision*^*#*^	12 (10.9)	1 (2.5)	13 (8.7)
	*Internal hernia*	8 (7.3)	3 (7.6)	11 (7.4)
	*Hiatal hernia*	5 (4.5)	3 (7.6)	8 (5.4)
	*Trocar hernia*	4 (3.6)	0	4 (2.7)
	*Incisional hernia*	2 (1.8)	0	2 (1.3)
	*Abdominal wall abscess*	0	1 (2.5)	1 (0.7)
	*Marginal ulcer*	1 (0.9)	0	1 (0.7)

^*^All eroded rings were removed via endoscopy. Of the 5 patients with dysphagia, 4 had their ring removed and one ring was replaced. In all other ring-related complications, the ring was replaced

^#^In 3 patients pouch revision was performed due to weight recurrence and in 1 due to stenosis. In the other 9 patients, the indication for operation was either dysphagia (5 patients) or hiatal hernia (4 patients)

For both * and #: if not endoscopically, the complications were solved surgically

**Table 5:** Complications between 6 and 10 years post-operative

Complications 6–10 years		Primary (%)	Conversional (%)	Total (%)
Patients with complications		13 (11.8)	5 (12.8)	18 (12.1)
Complications		27	9	36
Ring related*		5 (4.5)	3 (7.7)	8 (5.4)
	*Erosion*	0	2 (5.1)	2 (1.3)
	*Slippage*	3 (2.7)	0	3 (2.0)
	*Stenosis*	1 (0.9)	1 (2.6)	2 (1.3)
	*Broken ring*	1 (0.9)	0	1 (0.7)
Ring unrelated		22 (20.0)	6 (15.4)	28 (18.8)
	*Pouch revision*	11 (10.0)	1 (2.6)	12 (8.1)
	*Internal hernia*	4 (3.6)	4 (10.3)	8 (5.4)
	*Hiatal hernia*	4 (3.6)	1 (2.6)	5 (3.4)
	*Trocar hernia*	1 (0.9)	0	1 (0.7)
	*Incisional hernia*	1 (0.9)	0	1 (0.7)
	*Marginal ulcer*	1 (0.9)	0	1 (0.7)

^*^Only the eroded rings were removed, via endoscopy. All other rings were replaced surgically (4.0%)

In the primary group, no rings were removed between 6 and 10 years post-operative. However, in 5 patients, the ring was repositioned or replaced with a new silicone ring for the reasons mentioned in Table 4 and 5. For the conversional group, 2 patients had their ring removed due to erosion of the ring. These two rings were both removed endoscopically.

## Discussion

### Weight Loss

The present study demonstrates that 10 years after RYGB weight loss can be maintained in a high percentage of patients with the use of a silicon ring. The study by Margo et al. does describe the banded RYGB; however, the follow-up percentage is only 11.6. Therefore, the only other study thus far with sufficient long-term follow-up results describing the 10-year follow-up of a BRYGB is the study by Awad et al. [9, 11]. They did show a maintenance of weight loss in their banded patient group similar to our study. However, 80% of the BRYGB were performed as open surgery.

When comparing these results to the results of non-BRYGB, Awad et al. showed significantly more weight loss in the banded group after 10 years and, interestingly, a steady decline in total weight loss starting from around 2 years post-operative until at least 10 years post-operative in the non-banded group. Other long-term follow-up studies on non-BRYGB presented similar evolution of weight loss over the years [12–14].

### Weight Recurrence

After the initial weight loss from bariatric surgery, most patients will suffer weight recurrence. There is no consensus about what amount of weight recurrence is clinically significant yet. For instance, a review by El Ansari et al. described eight different definitions of weight recurrence used in bariatric studies [15]. Voorwinde et al. underline these outcomes when concluding standardized weight recurrence definitions are not available yet and should be defined using clinical outcome measures such as quality of life [16].

In the current study, different percentages of TWL are used to group the study patients into weight recurrence yes or no. These different percentages are described at 5 and 10 years to give a clear overview of the number of people with weight recurrence at these time points. When comparing these results to the existing literature concerning non-BRYGB, a clear difference can be noticed [13, 17, 18]. For instance, the study by Duvoisin et al. shows 43.8% of patients with more than 25% weight recurrence compared to only 5.1% of patients with more than 20% weight recurrence in the current study [18].

### Associated Medical Problems

The ultimate goal of bariatric surgery is the improvement of the health of the patient and increasing quality of life. In the current study, data on four diseases associated with obesity were recorded (type 2 diabetes, OSAS, hypercholesterolemia, and hypertension). After 10 years, a significant decrease in these associated medical problems can be objectified when compared to baseline. However, when comparing the 5- and 10-year results, an increase can be seen in the number of patients with diabetes mellitus and dyslipidemia. Unfortunately, studies researching RYGB that do describe associated medical problems after 10 years only present the 10-year endpoint results and not the results between operation and the 10-year endpoints [14, 19]. Comparing the results over time of existing literature with the current study is therefore not possible.

A comparison with the 10-year results of an RYGB can be made. Higa et al. showed a 67–86% reduction in associated medical problems at 10 years. However, this was only calculated in 21% of the total study group since the rest was lost to follow-up. Mehaffey et al. also described a statistical difference between the pre-operative prevalence of associated medical problems compared to 10 years post-operative. Unfortunately, it is unclear how many patients are lost to follow-up at 10 years in this study [18, 20–22]. These results are however comparable to this study’s results with a reduction of 42–75% in associated medical problems after 10 years post-operative with a 10-year follow-up of 60–64.5%.

### Complications

A concern when using a silicon ring is the risk of ring-related complications. The results of this study show that with primary surgery the risk of ring-related complications in 10 years is 12.7%, with 8% risk in the first 5 years [7]. Unfortunately, there are no other studies clearly describing all forms of complications after 10 years. Different studies on BRYGB present a 2–4% risk of ring-related complications after 5 years [5, 6]. A possible explanation for the difference in complication rates is that the patients in the current study were part of the early experience with the BRYGB in our center. As can be seen in a later cohort, the complication rates decreased [5]. Although different complications can occur when using a silicone ring, most of these complications can easily be resolved by either a reoperation or even endoscopically as can be seen in this study’s population.

### Comparison to Literature

The current study solely describes the results of the BRYGB. In addition to the above-described comparisons between the BRYGB and the RYGB, Table 6 presents an overview of different studies with their 10-year results concerning weight loss and complications.

**Table 6:** Data of the current study compared to existing literature concerning total weight loss (%TWL) and complication rates

Article	*N* at start	FU rate at 10 years	%TWL at 10 years	Complications
Current study	110	71.8%	30 (SD 11.0)	22.7%
Salminen, JAMA Surg (2022) [23]	119	79.8%	26.9 (95% CI 25.6–28.2)	18.5%
Liagre, SOARD (2022) [24]	535	74.6%	27.3 (SD 12.3)	32.9%*
Gorecki, SAGES Oral (2021) [13]	576	25.2%	28.2 (SD 11.6)	NA
Higa, SOARD (2011) [20]	242	26.9%	28.8 (SD 11.3)	27.3%

*FU* follow-up, *%TWL* total weight loss

^*^Not all complications were clearly described; therefore, there might be some missing complications in this total

### Strengths and Limitations of the Study

Due to the retrospective character of the study, data which were not recorded in the past are not available to us. Therefore, no quality-of-life information was available for analysis.

Although this was a retrospective data study, the loss to follow-up percentage was low when compared to other studies with 10 or more years of follow-up. However, it is important to mention that part of our data was self-reported, which can affect the quality of the data. At 10 years, the combination of patient files and self-reported data resulted in a follow-up percentage of 71.8 in the primary group and 53.8 in the conversional group.

## Conclusion

The results of this study suggest that the addition of a silicone ring to the Roux-en-Y gastric bypass results in the maintenance of weight loss 10 years post-operative. Furthermore, the number of associated medical problems was significantly reduced and the number of patients with long-term complications was low. When comparing these data with the available long-term follow-up data from non-BRYGB in literature, we can conclude that a banded Roux-en-Y gastric bypass has a more sustained effect on weight loss, especially in the long run.

## Data Availability

The data underlying this article will be shared on reasonable request to the corresponding author.
